# The effect of exercise on intramyocellular acetylcarnitine (AcCtn) concentration in adult growth hormone deficiency (GHD)

**DOI:** 10.1038/s41598-019-55942-w

**Published:** 2019-12-19

**Authors:** Fabian Meienberg, Hannah Loher, Julie Bucher, Stefan Jenni, Marion Krüsi, Roland Kreis, Chris Boesch, Matthias Johannes Betz, Emanuel Christ

**Affiliations:** 1 Endocrinology & Diabetology, Kantonsspital Baselland, Liestal, Switzerland; 2 Innere Medizin, Kantonsspital, St. Gallen, Switzerland; 3 EndoDia Praxis, Biel, Switzerland; 4 Praxis Endokrinologie Diabetologie Bern, Bern, Switzerland; 5 Praxis Endokrinologie & Diabetologie, Zürich Unterland, Embrach, Switzerland; 6 Departments of Biomedical Research and Radiology, University Bern, Bern, Switzerland; 7 Endocrinology, Diabetes & Metabolism, University Hospital Basel and University of Basel, Basel, Switzerland

**Keywords:** Pituitary diseases, Predictive markers

## Abstract

To cover increasing energy demands during exercise, tricarboxylic cycle (TCA) flux in skeletal muscle is markedly increased, resulting in the increased formation of intramyocellular acetylcarnitine (AcCtn). We hypothesized that reduced substrate availability within the exercising muscle, reflected by a diminished increase of intramyocellular AcCtn concentration during exercise, might be an underlying mechanism for the impaired exercise performance observed in adult patients with growth hormone deficiency (GHD). We aimed at assessing the effect of 2 hours of moderately intense exercise on intramyocellular AcCtn concentrations, measured by proton magnetic resonance spectroscopy (^1^H-MRS), in seven adults with GHD compared to seven matched control subjects (CS). Compared to baseline levels AcCtn concentrations significantly increased after 2 hours of exercise, and significantly decreased over the following 24 hours (ANOVA p for effect of time = 0.0023 for all study participants; p = 0.067 for GHD only, p = 0.045 for CS only). AcCtn concentrations at baseline, as well as changes in AcCtn concentrations over time were similar between GHD patients and CS (ANOVA p for group effect = 0.45). There was no interaction between group and time (p = 0.53). Our study suggests that during moderately intense exercise the availability of energy substrate within the exercising muscle is not significantly different in GHD patients compared to CS.

## Introduction

Adult growth hormone deficiency (GHD) is characterized by impaired exercise performance. The aerobic capacity (VO_2 max_) in adults with GHD is reduced by 17% to 27% compared to values predicted for age, gender and height^1^, and there is strong evidence that growth hormone (GH) replacement improves exercise performance in GHD patients^2^. Several potential mechanisms have been postulated to explain this finding^3^: e.g. anabolic effects of GH on skeletal muscle and effects on the cardiovascular system by increasing preload^4^ and decreasing afterload^5^ as well as increasing red cell mass^4^. Furthermore, and as explained in more detail below, reduced availability of energy substrate (in particular free fatty acids (FFA)) within the exercising muscle may play an important role^3^.

**Figure 1:**
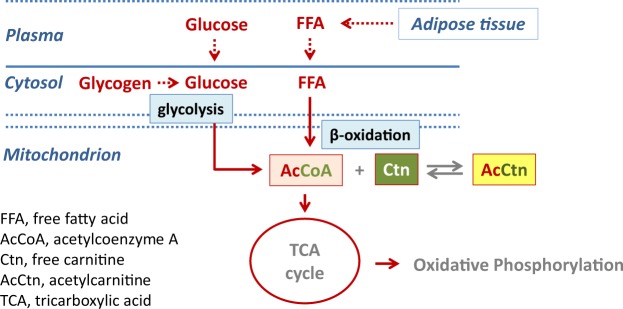
Formation of acetylcarnitine (AcCtn) and energy supply in the exercising skeletal muscle. During exercise, glycolysis and beta-oxidation are markedly increased, and the production of acetyl-Coenzyme A (AcCoA) exceeds its entry into the tricarboxylic acid (TCA) cycle. To avoid accumulation of AcCoA, acetyl groups are transferred to free carnitine (Ctn) forming Acetylcarnitine (AcCtn). Thus, the increase in AcCtn production is directly related to the formation of AcCoA. With prolonged exercise intracellular energy stores decrease, and ATP production increasingly depends on the supply of glucose and free fatty acids (FFA) from the systemic circulation.

Skeletal muscle contraction and relaxation depend upon energy derived from adenosine triphosphate (ATP). With prolonged exercise intracellular energy stores in skeletal muscle (i.e. glycogen and intramyocellular lipids (IMCL)) decrease, and ATP production increasingly depends on the supply of glucose and FFA from the systemic circulation^6^ (Fig. 1). These systemic metabolic adaptations are regulated by hormones such as cortisol, catecholamines and growth hormone (GH)^7^. In the fasting state GH is known as a potent lipolytic hormone, stimulating the release and oxidation of FFA from adipose tissue^8^. The lipolytic effect of GH has been confirmed in studies with GHD patients, demonstrating that GH replacement therapy increases lipolysis and tissue uptake of FFA^9,10^, as well as fat oxidation^11^ during exercise. However, these studies used stable isotope technique to study FFA flux, and this method does not provide information on the specific site of FFA uptake. Thus, it remains unproven whether these findings really translate into reduced substrate availability within exercising skeletal muscle, and possibly thereby limiting exercise performance.

In contrast to isotope studies, the measurement of skeletal muscle acetylcarnitine (AcCtn) concentrations by proton MR spectroscopy (^1^H-MRS) provides information on energy metabolism specifically from within the exercising muscle, and due to its non-invasive nature, this procedure widely obviates the need for repeated muscle biopsies.

The formation of skeletal muscle AcCtn serves as a marker for intramuscular ATP production via oxidative phosphorylation. In exercising skeletal muscle, changes in AcCtn concentrations are directly related to changes in acetyl-CoA (AcCoA) concentrations^12–14^. AcCoA results from glycolysis and from beta-oxidation of FFA. It enters the tricarboxylic acid (TCA) cycle (also known as ‘citric acid cycle’ or ‘Krebs cycle’), which provides the substrates for ATP production via oxidative phosphorylation^15^ (Fig. 1). During exercise - with the increasing demand for ATP production - TCA cycle flux in skeletal muscle is dramatically increased (up to 100-fold). This process is associated with increased concentrations of various TCA cycle intermediates, including AcCoA^16,17^. Finally, the concentration of AcCoA exceeds the capacity of flow into the TCA cycle^13,18^. To avoid accumulation of AcCoA and thus depletion of free CoA, acetyl groups are transferred from AcCoA to free carnitine, forming AcCtn in a reversible reaction^19^. Hence, free carnitine acts like a sink for excess AcCoA (Fig. 1), and the increase in AcCtn concentration reflects the formation of AcCoA.

The missing lipolytic action of GH in adult GHD may be associated with impaired availability of intramuscular energy substrate (in particular FFA) during prolonged aerobic exercise. This would result in reduced intramuscular beta-oxidation of FFA, and thus reduced formation of AcCoA. Finally, this would result in a blunted increase of intramyocellular AcCtn concentration during exercise.

We, therefore, hypothesize that (1) intramyocellular AcCtn levels increase during aerobic physical exercise and decrease thereafter. We expect that (2) intramyocellular AcCtn levels increase less in GHD patients compared to control subjects (CS).

## Methods

We performed a prospective single-center trial at the University Hospital of Bern, Switzerland. The trial was approved by the local review board (Ethics Commission of the Canton of Bern, Switzerland) and all subjects gave written informed consent. The study was conducted according to the declaration of Helsinki, the guidelines of good clinical practice and Swiss health laws on clinical research. This trial was registered under the following clinical trial number: NCT01467193.

### Study participants

The current paper presents additionally available data of our previous report^20^; and we did not perform a specific power calculation for the main endpoint reported in the present paper.

Briefly, seven male patients with severe GHD and seven male sedentary CS matched for age, BMI and waist circumference were included. Severe GHD was defined according to the current guidelines^21^. All of our participants were non-institutionalized, in good general health, and capable to exercise on a bicycle ergometer for two hours. GHD patients were included if they had been under stable conventional hormone replacement therapy - except for GH - as needed (i.e. glucocorticoids, thyroxin and testosterone) for at least 6 months. Inclusion criteria for CS were no regular physical activity (<60 minutes/week), and no regular medication intake, as reported in a health questionnaire.

Exclusion criteria were (former or present) ACTH- or GH-secreting pituitary adenoma, severe cardiovascular disease, diabetes mellitus, abnormal liver or renal function, active neoplasia, hemophilia, and therapy with drugs known to affect lipid or glucose metabolism or inability to exercise.

### Study protocol

All the participants attended the research unit for three visits. The first and second visit were scheduled within 7–10 days. The third visit was scheduled on the day following the second visit.

#### Determination of baseline characteristics and VO_2max_ (visit 1)

Participants attended the endocrine investigation unit after an overnight fast and having restrained from physical activity for 72 hours before the test. End-expiratory waist circumference was measured with a measuring tape placed on a horizontal plane at the level of the iliac crest. A blood sample was taken by venipuncture (Insulin, Glucose, Free fatty acids (FFA), total Cholesterol, LDL-Cholesterol, HDL-Cholesterol, Triglycerides, Thyroidea-stimulating-hormone (TSH), free Thyroxin (fT4), free Triiodothyronine (fT3), total Testosterone, Follicle-stimulating hormone (FSH), Luteinizing hormone (LH), Cortisol, Insulin-like growth factor 1 (IGF-1). Maximal aerobic exercise capacity was determined during an incremental workload test on a bicycle ergometer as previously reported^22^.

#### Two-hour physical exercise on a bicycle at 50% of VO_2max_ (visit 2 + 3)

GHD patients and CS attended the hospital after an overnight fast. In the morning, they received a standardized light meal (380 kcal) consisting of 2 deciliter of orange juice, yoghurt, curd and cereals. Subsequently, between 1:30 pm and 3:30 pm, patients and CS exercised on a bicycle for 2 hours at 50% of the pre-assessed VO_2max_. This protocol has been shown to reliably deplete IMCL stores, and to increase systemic FFA concentrations, indicating systemic lipolysis^22,23^. In the GHD patients hydrocortisone replacement therapy was administered as needed 30 minutes before physical exercise.

Skeletal muscle AcCtn concentrations were measured by ^1^H-MRS before (0 h, Baseline) and immediately after exercise (2 h). Visceral (VAT) and subcutaneous (SCAT) adipose tissue were separately assessed before the exercise using MR images (see below). During the exercise, blood samples were taken from an indwelling catheter.

In the evening after the exercise, the subjects had a standardized meal consisting of 2 deciliter of orange juice, yoghurt, curd and cereals. The following morning, subjects received a standardized breakfast (identical amount of calories and content as on the day before).

After re-attending the research unit, another blood sample was taken and 24 hours after the exercise a third measurement of skeletal muscle AcCtn concentrations was performed (24 h).

### Biochemical analysis

Serum IGF-1 concentrations were determined using a chemiluminescent immunometric assay (Immulite, Siemens, Zürich, Switzerland). Cortisol concentrations were measured with Modular (Roche, Rotkreuz, Switzerland). Total testosterone concentrations were analyzed using Beckman Coulter RIA (IM1119). Plasma glucose concentration levels were measured by an enzymatic hexokinase method (Modular P800, Roche). Insulin concentrations were measured with an immunoassay (Architect, Abbott, Baar Switzerland). Insulin sensitivity was estimated using homeostasis model assessment (HOMA, glucose × insulin/22.5)^24^. FFA concentrations were determined using a commercially available kit (Wako Chemicals, Dietikon Switzerland). Isolation of lipoprotein subfractions, including VLDL and measurements of VLDL-TG were performed as previously described^25^.

### MR measurements

All magnetic resonance examinations were performed on a Siemens Verio 3 T MR system as previously described^22^.

#### Skeletal muscle AcCtn concentration

For ^1^H-MRS of skeletal muscle, the body transmit coil and a local surface receive coil array were used in combination with the manufacturer’s standard PRESS sequence (echo time TE 30 ms, repetition time TR 3 s) to record the signal from the region of interest (ROI, 9 × 9 × 18 mm^3^) in vastus intermedius (prescribed on axial spin echo MR images for repositioning after exercise based on anatomical markers). A spectrum with water presaturation (50 Hz bandwidth), 96 averages and the transmit frequency centred at 1.5 ppm was acquired. For quantification and eddy current correction a fully relaxed spectrum of water was also recorded (no water suppression, one acquisition, centred at 4.7 ppm, 76% assumed water content for quantification). Fitting of the metabolite and IMCL resonances was performed in FiTAID^26^ using linear combination model fitting for the metabolites creatine (Cr_tot_, creatine plus phosphocreatine), trimethyl ammonium compounds (TMA), taurine, AcCtn, IMCL and extramyocellular lipids (EMCL). Prior knowledge was used to stabilize the fit, in particular of AcCtn superimposed on broader peaks of EMCL and IMCL (fixed frequency differences between resonances of Cr_tot_, TMA, taurine and AcCtn). Further constraints for signal modeling included: uniform phase, fixed Lorentz widths and a Gaussian width that was allowed to vary per spectrum to adapt to specific line broadenings. Furthermore, the dipolar peak patterns of taurine and Cr_tot_ were adapted per subject and then kept constant for all three spectra recorded. The subpatterns of the lipid spectra were adapted on summed spectra and then kept constant for individual fits.

Repositioning of the ROIs in follow-up examinations was based on two steps. Coarse repositioning was achieved by placing the leg at the same position relative to the coil using the palpated inferior end of the patella. Careful comparison with MR images from the first exam provided guidance for fine repositioning based on visible fasciae that were carefully evaded in the first examination as well as blood vessels.

#### SCAT and VAT

SCAT and VAT were assessed as previously described^23^ using T_1_-weighted MR images and a point-counting method^27^. Fat compartments were assessed for volume, and then converted to mass (kilograms). Lean body mass was calculated by difference.

### Statistical analysis

Data are expressed as mean ± standard deviation (SD). Baseline parameters and AcCtn concentrations of GHD patients and CS at baseline were compared using the unpaired t-test. Changes in AcCtn concentrations between different time points were analyzed by repeated measures ANOVA followed by Sidak’s test for multiple comparisons. Associations between changes in AcCtn concentrations and various parameters were analyzed by Pearson correlation.

All statistical analyses were performed with GraphPad Prism Version 8.2 (GraphPad Inc., La Jolla, CA). A p-value of < 0.05 was considered statistically significant.

## Results

### Participants’ clinical and biochemical baseline characteristics

Seven male subjects, aged 26 to 58 years, with severe GHD and seven sedentary CS a priori matched for age, BMI and waist circumference were included in the study.

The characteristics of the GHD subjects, including causes of GHD, treatment of the underlying disease, and necessity of hormone replacements are summarized in Table 1. Substitution of corticotropic, gonadotropic and thyreotropic axis was established in 4, 6, and 5 patients, respectively. The clinical and biochemical characteristics of all participants are summarized in Table 2. Patients and CS were statistically not different with regard to the matching criteria, i.e. age, BMI, and waist circumference. Mean BMI was 26.7 kg/m^2^ (range 20.2–31.8 kg/m^2^) and 27.0 kg/m^2^ (range 23.3–35.2) in GHD patients and CS, respectively. Visceral and subcutaneous fat mass were similar in the two groups, as were parameters for lipid and glucose metabolism, cortisol and testosterone levels. As expected, IGF-1 levels and VO_2max_ were significantly lower in the GHD patients compared to the CS. Individual baseline data are presented in Supplementary Table 1.

**Table 1:** Clinical characteristics of the seven study participants with growth hormone deficiency (GHD).

Age	Diagnosis	Treatment	Duration of hypopit. (years)	Hormone deficiencies				
				GH	ACTH	FSH/LH	TSH	ADH
26	Medulloblastoma	RT	10	x			x	
37	Pituicytoma	Surgery, RT	3	x	x	x	x	x
47	NFPA	Surgery, RT	8	x		x		
48	Rathke-Cleft-Cyst	Surgery	18	x	x	x	x	x
56	Idiopathic Hypopit.	—	46	x		x		
56	NFPA	Surgery, RT	21	x	x	x	x	
58	Plasmacytoma	RT	15	x	x	x	x	

hypopit. = hypopituitarism; NFPA = non-functioning pituitary adenoma; RT = radiotherapy.

GH = growth hormone; ADH = antidiuretic hormone.

**Table 2:** Clinical and biochemical characteristics fat mass and exercise capacity of the GHD patients and CS participating in the study.

	GHD (n = 7)	CS (n = 7)	p-values
Age (years)	46.9 ( ± 11.7)	39 ( ± 12.6)	0.25
Weight (kg)	80.9 ( ± 15.3)	83 ( ± 14.5)	0.80
LBM (kg)	64.6 ( ± 11.3)	64.8 ( ± 9.4)	0.97
BMI (kg/m^2^)	26.7 ( ± 3.8)	27.0 ( ± 4.1)	0.88
Waist (cm)	93.3 ( ± 12.8)	91.3 ( ± 13.8)	0.78
*Body fat*			
SAT (kg)	13.8 ( ± 4.3)	13.5 ( ± 5.9)	0.91
VAT (kg)	3.5 ( ± 1.5)	2.2 ( ± 1.8)	0.22
*Exercise capacity*			
VO_2max_ (ml*kg^−1^ min^−1^)	30.5 ( ± 6.2)	42.8 ( ± 10.9)	**0.03**
*Fasting biochemical parameters*			
Glucose (mmol/L)	4.9 ( ± 0.4)	5.4 ( ± 1.0)	0.19
Total Cholesterol (mmol/L)	5.4 ( ± 1.1)	5.8 ( ± 1.0)	0.46
HOMA	2.4 ( ± 1.5)	2.8 ( ± 2.3)	0.70
LDL-Cholesterol (mmol/L)	3.7 ( ± 1.1)	3.9 ( ± 0.8)	0.72
Triacylglycerol (mmol/L)	1.4 ( ± 0.7)	1.3 ( ± 1.0)	0.84
VLDL-TG (mmol/L)	0.83 ( ± 0.70)	0.76 ( ± 0.75)	0.85
FFA (mmol/L)	0.5 ( ± 0.1)	0.7 ( ± 0.4)	0.20
Cortisol (nmol/L)	468.7 ( ± 172.5)	433.3 ( ± 113.9)	0.68
IGF-1 (ng/ml)	80.2 ( ± 47.5)	139.5 ( ± 33.2)	**0.02**
Testosteron (nmol/L)	22.6 ( ± 11.4)	17.7 ( ± 4.8)	0.31

GHD = patients with growth hormone deficiency; CS = control subjects.

waist = waist circumference; LBM = lean body mass; SAT = subcutaneous adipose tissue mass; VAT = visceral adipose tissue mass; VO_2max_ = maximal oxygen uptake; HOMA = HOMA-Index: fasting Insulin*fasting Glucose*22,5^−1^; FFA = free fatty acids.

Values are mean ( ± SD), bold p-values: statistically significant (p < 0.05).

### Skeletal muscle AcCtn concentrations

**Figure 2:**
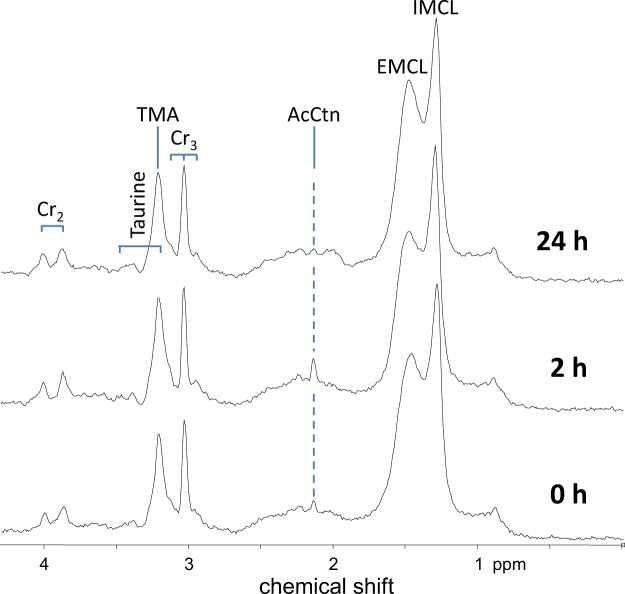
Representative spectra of a control subject. The AcCtn peak, i.e. the resonance of the acetyl group of the molecule that is visible as a singlet at 2.1 ppm, documents clearly elevated tissue content 2 hours after aerobic exercise, and decreases over the following 24 hours. Spectral quality and its reproducibility is excellent, as readily documented by equal EMCL contributions (identical region of interest) and identical peak splitting for the Cr_2_ peak (identical muscle fiber orientation in the magnet) at all three time points. AcCtn = acetylcarnitine; IMCL = intramyocellular lipids; EMCL = extramyocellular lipids; TMA = trimethyl ammonium compounds; Cr_2_, Cr_3_ = Creatine.

Representative spectra obtained from a control subject at all three time points are presented in Fig. 2. Intramyocellular AcCtn concentrations at baseline and changes over time are shown in Table 3. With regard to the entire study population (n = 14, GHD and CS taken together) baseline levels of AcCtn concentrations significantly increased after 2 hours of exercise, and significantly decreased over the following 24 hours (ANOVA p for effect of time = 0.0023). The same effect has been observed within each of the two groups (GHD and CS), although the effect did only reach borderline significance (ANOVA p = 0.067 in GHD, p = 0.045 in CS). AcCtn concentrations at baseline (p = 0.90), as well as changes in AcCtn concentrations over time were similar between GHD patients and CS (ANOVA p for group effect = 0.45). There was no interaction between group and time (p = 0.53).

**Table 3:** Intramyocellular acetylcarnitine (AcCtn) concentrations (mmol/kg) at baseline, after 2 hours of exercise, and 24 hours post-exercise.

	all study participants (n = 14)	GHD (n = 7)	CS (n = 7)
Baseline	0.35 ( ± 0.48)	0.33 ( ± 0.50)	0.38 ( ± 0.51)
2 hours	1.66 ( ± 1.47)	1.30 ( ± 1.19)	2.01 ( ± 1.72)
24 hours	0.75 ( ± 0.78)	0.63 ( ± 0.43)	0.88 ( ± 1.05)
*p-value (time course) (ANOVA)*	*p* = *0.0023*	*p* = *0.067*	*p* = *0.045*
*p-value for group effect (ANOVA)*	*P* = *0.45 No significant interaction between group and time*		

GHD = patients with growth hormone deficiency; CS = control subjects.

Values are mean ( ± SD). Differences between time-points were tested after repeated measures ANOVA by Sidak’s multiple comparison test versus baseline.

AcCtn concentrations at baseline (unpaired t-test, p = 0.90), as well as changes over time (ANOVA) were similar between GHD and CS.

**Figure 3:**
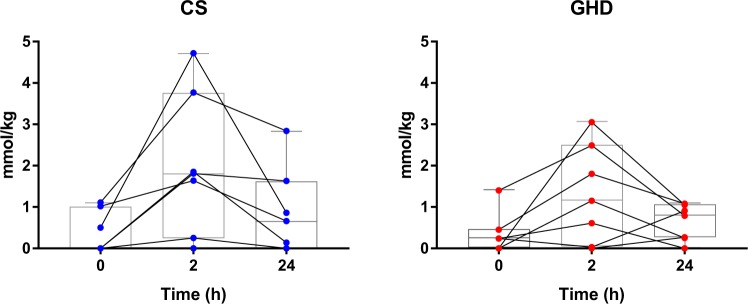
Changes in intramyocellular acetylcarnitine (AcCtn) concentrations of seven patients with GHD (right) and seven CS (left). Intramyocellular AcCtn concentrations were measured by proton magnetic resonance spectroscopy (^1^H-MRS) at baseline (0 h), 2 hours after moderately intense exercise (2 h), and 24 hours post-exercise (24 h). Changes of AcCtn concentrations over time did not significantly differ between GHD patients and CS (ANOVA p for group = 0.45). Bars, boxes and whiskers represent median, IQR and range. GHD = growth hormone deficiency, CS = control subjects.

Individual AcCtn concentrations over time in GHD patients and CS are shown in Fig. 3. Individual outcome data are presented in Supplementary Table 1. As there was considerable individual variation in the change of AcCtn concentrations over time, we repeated the ANOVA after exclusion of participants who did not show the expected increase of AcCtn with exercise (GHD participants 2 + 5, CS participant 5; Supplementary Table 1). As expected, the effect of time became more significant (p = 0.0007), whilst there was still no significant effect with respect to group (p = 0.53).

With regard to the entire study population (n = 14) changes in AcCtn concentrations over time did not correlate with the following baseline parameters: age, BMI, VO_2max_, HOMA index, lean body mass, total fat mass, VAT, SCAT (Supplementary Table 2).

## Discussion

The main findings of the current study can be summarized as follows: (1) two hours of moderately intense exercise result in a transitory increase in skeletal muscle AcCtn and a decrease 24 h thereafter, and (2) GHD does not significantly affect changes in skeletal muscle AcCtn during and after exercise.

The current finding of an increase of AcCtn after a 2 h moderate intensity aerobic exercise is consistent with previous studies that assessed AcCtn using repetitive skeletal muscle biopsies^14,19,28^. Importantly, studies using the non-invasive tool, namely ^1^H-MRS, confirmed the exercise-induced increase in AcCtn during short-term, high intensity^29–32^ and prolonged, moderately intense exercise^30,33,34^. This indicates that ^1^H-MRS is a reliable tool to assess changes in intramyocellular AcCtn concentration during exercise.

Our data demonstrate that 24 hours after exercise, intramuscular AcCtn returned towards baseline levels. To our knowledge, these measurements are a new finding indicating that the availability of AcCtn is – like intramyocellular lipids – flexible with a consistent increase immediately after exercise and a decrease thereafter. It has previously been shown that 10–15 minutes after the cessation of short-term high intensity exercise AcCtn levels start to decrease but remain elevated compared to pre-exercise values^13,29^. It is conceivable that differences in exercise protocol (high-intensity vs moderate intensity) and time point of measurement of intramuscular AcCtn influence the results.

Our study has demonstrated that with the exercise protocol applied, GHD does not significantly impact on changes in skeletal muscle AcCtn, and thus the formation of AcCoA. As we did not measure exhaled air during the exercise (to determine the respiratory exchange ratio), we cannot determine whether the source of AcCoA (glycolysis vs. beta-oxidation of FFAs) differed between GHD patients and CS. However, overall mitochondrial ATP production does not seem to be impaired in GHD patients. As expected, GH concentrations did not increase during exercise in GHD patients, suggesting reduced GH-induced systemic lipolysis. However, the systemic availability of FFA (expressed as AUC of FFA) was similar in GHD patients and CS, only peak serum FFA concentrations at the end of physical exercise were lower in GHD patients compared to CS (full data shown in our previous report^20^). This suggests that there are redundant endocrine systems affecting lipolysis (catecholamines, cortisol) which are sufficient to guarantee an adequate energy supply during moderate intensity exercise. In contrast, in another study assessment of systemic lipolysis using stable isotope technique indicated that lipolysis during exercise is reduced in GHD patients^9^. However, in this study a very different exercise protocol (15–30 min exercise until exhaustion) has been applied, which may explain the dissimilar results. Taken together, impaired exercise capacity seen in GHD patients is rather not related to significantly impaired lipolysis, during moderate intensity exercise.

The strength of our study lies in the a priori matching of CS for age, BMI, and waist circumference as part of the study design. Importantly, the matching for waist circumference, resulting in similar amounts of SCAT and VAT, did allow us to specifically examine short-term metabolic adaptions to exercise in GHD - independently of the characteristically increased fat mass observed in these patients. In addition, insulin concentrations and insulin sensitivity - important regulatory factors of lipolysis^35^ - were not statistically different between GHD patients and CS. The strict standardization of diet and exercise during the study (as potential factors influencing metabolism in the exercising muscle) is a further, essential strength of our study.

Still, there are several limitations in this study: (1) we included only male GHD patients and CS in order to avoid possible sex hormone induced heterogeneity in lipid metabolism. (2) It is possible that due to the small number of participants included, subtle differences between GHD patients and CS may have escaped detection, as suggested by the fact that numerically the increase in AcCtn with exercise was more pronounced in CS than in GHD patients. This restriction was mainly due to the sophisticated and time-consuming investigations and the strict matching criteria of CS with the patients with GHD. (3) AcCtn levels were measured immediately *after* and, strictly speaking, not *during* exercise. Differences in the dynamics of declining AcCtn levels after exercise could have blunted an inherent difference of AcCtn levels during exercise. However, as our protocol consisted of steady-state exercise we assume that post-exercise AcCtn concentrations provide a reasonable indicator of substrate availability *during* exercise.

In conclusion, based on measurements of AcCtn concentrations, our study suggests that during exercise the availability of energy substrate within the exercising muscle is not significantly different in GHD patients compared to CS. The redundant metabolic actions of other hormones (catecholamines, cortisol) probably compensate for the reduced lipolytic activity in GHD. The known effects of GH on skeletal muscle and on the cardiovascular system appear to be more important to improve the impaired exercise capacity than GH-induced metabolic effects on energy substrate availability.

## Supplementary information

Supplementary Table 1

Supplementary Table 2

## Data Availability

The datasets generated or analyzed during the current study are available from the corresponding author on reasonable request.
